# Preemptive analgesia with parecoxib sodium alleviates short-term pain after endoscopic submucosal dissection for early esophageal cancer and precancerous lesions: a single-center, double-arm, and randomized controlled study

**DOI:** 10.3389/fonc.2026.1858955

**Published:** 2026-08-14

**Authors:** Xiao-Bo Liu, Hai-Tao Shen, Zi-Mei Chen, Wen Xu, Shu Jin, Yuan-Jun Gao

**Affiliations:** 1 Department of Gastroenterology, Taihe Hospital, Hubei University of Medicine, Shiyan, Hubei, China; 2 Department of Respiratory, Yangquan First Hospital, Yangquan, Shanxi, China

**Keywords:** early esophageal cancer, endoscopic submucal dissection, parecoxib sodium, postoperative pain, precancerous lesions, preemptive analgesia

## Abstract

**Introduction:**

Endoscopic submucosal dissection (ESD) is the preferred treatment for early esophageal cancer and precancerous lesions. Postoperative pain is a prevalent but often overlooked issue.This study investigated the analgesic efficacy and safety of preemptive parecoxib sodium in patients undergoing ESD for early esophageal cancer and precancerous lesions.

**Methods:**

Patients scheduled for ESD under general anesthesia were enrolled and randomly allocated to the medication group (intravenous parecoxib sodium 30 minutes prior to surgery) or the control group (4 mL intravenous normal saline 30 minutes before the procedure). Clinical data, including perioperative serum levels of high-sensitivity C-reactive protein, IL-6, complete blood count, and biochemical parameters, were collected. Postoperative pain intensity was assessed using the visual analog scale (VAS) at 6, 12, 24, 72, and 120 hours after the procedure.

**Results:**

A total of 78 patients participated (40 in the parecoxib group, 38 in the control group). The two groups were comparable in baseline characteristics and preoperative inflammatory/nutritional indicators (all P > 0.05). At 6 and 12 hours post-operation, pain scores in the medication group were significantly lower than those in the control group (P < 0.05), while VAS scores at 24, 72, and 120 hours showed no significant differences between groups (P > 0.05). The frequency and total dosage of postoperative tramadol administration, as well as the incidence of adverse reactions, were comparable between the two groups (all P > 0.05).

**Conclusion:**

Preemptive analgesia with parecoxib sodium can alleviate early postoperative pain in patients with early esophageal cancer and precancerous lesions who undergo ESD for mucosal defects involving less than three-quarters of the esophageal circumference, without increasing adverse reactions or the subsequent use of analgesic drugs.

**Clinical trial registration:**

https://www.chictr.org.cn/showproj.html?proj=218572, identifier ChiCTR2400080433.

## 1 Introduction

Endoscopic submucosal dissection (ESD) is a minimally invasive procedure that preserves organ integrity and exhibits a low complication rate; hence, it as the preferred treatment for early esophageal cancer and precancerous lesions (1, 2). Despite the traditional view of post-ESD pain as negligible, recent studies indicate that it is prevalent. For instance, 98% of patients report pain following gastric ESD, particularly within 1–4 hours postoperatively (3–5). The incidence of moderate-to-severe pain after gastric ESD is 44.9% in patients using proton pump inhibitors (PPIs) and reaches 62.8% in those not using PPIs (6). A study from Maebashi Gunma University in Japan (7) found that 35.7% of patients experience chest pain after esophageal ESD. Therefore, enhanced postoperative pain management, particularly for esophageal ESD, warrants clinical attention.

The mechanisms and risk factors associated with post-ESD pain are not fully understood. Current evidence suggests that post-ESD pain is related to various factors, such as post-electrocoagulation syndrome, transmural burn and air leakage, heightened acid sensitivity, inflammatory tissue contraction, systemic inflammatory response, gas-related distension, and bile acid reflux (5–13). Insufficient pain management can result in procedure-related anxiety and may diminish patient compliance with essential future endoscopic surveillance, affecting long-term disease management. Consequently, effective postoperative analgesia is vital because not only it improves a patient’s immediate experience but also it fosters adherence to long-term follow-up, positively influencing overall prognosis.

Parecoxib is a selective cyclooxygenase-2 inhibitor (14) approved for the short-term management of acute perioperative pain, particularly for moderate-to-severe pain. Its administration can decrease the use of opioid analgesics and enhance postoperative recovery (15). This study aims to assess the effect of preemptive parecoxib on post-procedural pain in patients undergoing esophageal ESD and provide evidence that may contribute to the optimized perioperative management for this patient population.

## 2 Materials and methods

### 2.1 Study population

This single-center, double-arm, and prospective randomized study was conducted at Taihe Hospital from January 18, 2024, to December 1, 2024. Patients scheduled to undergo ESD for early esophageal cancer or precancerous lesions were enrolled.

### 2.2 Inclusion and exclusion criteria

The inclusion criteria were as follows: confirmed diagnosis of early esophageal cancer or precancerous lesions that meet ESD indications, established through preoperative white-light endoscopy, endoscopic ultrasonography, and magnifying endoscopy with narrow band imaging (NBI) staining, in conjunction with pathological examination; no history of allergy to anesthetic agents; and signed informed consent.

The exclusion criteria were as follows: lesions affecting ≥3/4 of the esophageal circumference, necessitating intraoperative or postoperative steroid administration for stricture prevention; pregnancy or lactation; significant hepatic or renal impairment, or severe cardiovascular or cerebrovascular diseases that render the patient unfit for the procedure; documented allergy or hypersensitivity to lidocaine, ropivacaine, parecoxib, other nonsteroidal anti-inflammatory drugs, or sulfonamides; preexisting neurological disorders, chronic pain syndromes, psychiatric conditions, or prolonged use of psychotropic or analgesic medications; and history of opioid misuse or substance dependence.

This trial adhered to the principles of the Declaration of Helsinki, was registered with the Chinese Clinical Trial Registry (Registration No.: ChiCTR2400080433), and approved by the ethics committee of Taihe Hospital (Approval No.: 2024KS07). All participants provided written informed consent.

### 2.3 Group allocation and management

**Figure 1:**
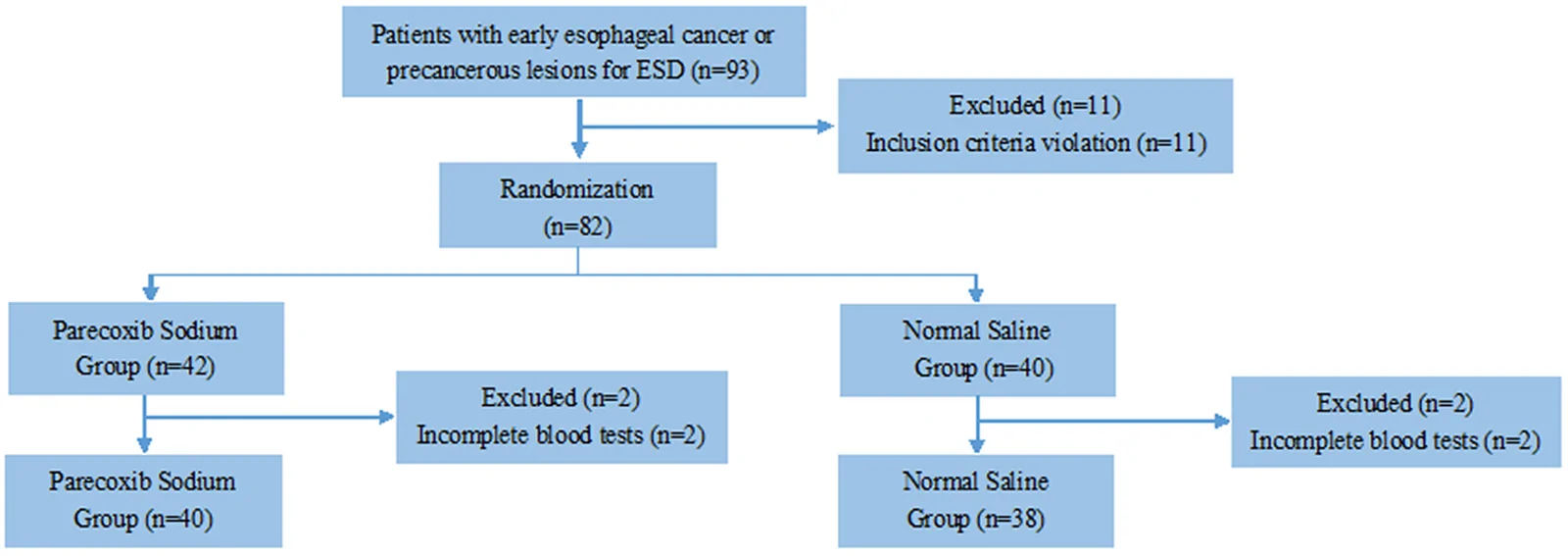
Schematic diagram of patient group allocation.

Patients were randomly assigned to one of two groups using a random number table: parecoxib sodium (group A) and control (group B). All patients received identical preoperative education and instructions, irrespective of their group assignment. After eligibility assessment by an anesthesiologist, the patients underwent esophageal ESD under combined intravenous-inhalation anesthesia in the endoscopy center operating room. Thirty minutes before the procedure, group A was administered an intravenous injection of 40 mg parecoxib sodium (diluted to 4 mL with normal saline), whereas group B received 4 mL of intravenous normal saline (**Figure 1**). The researchers conducted follow-up assessments and the participants were blinded to the group assignments.

### 2.4 ESD procedure and postoperative management

All ESD procedures were performed by seasoned endoscopists with senior professional designations. The surgical procedure adhered to a standardized sequence consisting of five essential steps: submucosal injection, lesion marking, circumferential incision, submucosal dissection, and wound management. The freshly resected specimen was fully extended to preserve tissue integrity. Its long diameter was measured and recorded, after which it was fixed in 10% neutral buffered formalin before being sent to the pathology department. The specimen was serially sectioned parallel to its long axis at 2–3 mm intervals, and 4–6 μm-thick tissue sections were prepared for routine staining. Pathological evaluation focused on the tumor’s maximum diameter, degree of differentiation, status of all margins (vertical and horizontal), depth of invasion, and the presence or absence of lymphovascular invasion. Immunohistochemical staining was employed when necessary to aid diagnosis.

The vital signs of the patients were monitored postoperatively. Proton pump inhibitors (PPIs) and intravenous nutritional support were administered. They were instructed to adhere to a strict fasting regimen (no food or water) for 2–3 days, and the diet was gradually changed from liquids to semiliquid, then to soft food, and finally to regular diet.

### 2.5 Study related definitions and concepts

1. Lesion Location: The location of a lesion is identified on the basis of esophageal anatomical segments, and the distance from the incisors is used as a reference point. The upper segment is 15–24 cm long; the middle segment, 24–32 cm; and, the lower segment, >32 cm.
2. Maximum Lesion Diameter: This parameter is measured on a formalin-fixed specimen and is defined as the greatest dimension, referred to as “length” in the expression “length × width”.
3. Resection Area: Assuming the resected specimen approximated an oval shape, the area (cm^2^) is calculated using the formula: (π × long diameter (cm) × short diameter (cm))/4 (16).
4. Depth of Invasion: Tumor infiltration depth is categorized through the Paris classification scheme. Type M indicates confinement to the mucosa, including the epithelium to the muscularis mucosae, whereas type SM denotes submucosal invasion that breaches the muscularis mucosae without involving the muscularis propria.
5. Histopathological Type: According to the WHO classification of digestive system tumors and clinical terminology, esophageal intraepithelial neoplasia is classified as either low-grade intraepithelial neoplasia or high-grade intraepithelial neoplasia. The most common histological type is squamous cell carcinoma, followed by adenocarcinoma and other rare types (17, 18).

### 2.6 Postoperative recording and measurements

Patient information was collected: demographic characteristics (name, sex, age, height, and weight), behavioral factors (history of smoking and alcohol consumption), Medical history (chronic diseases and prior surgical interventions). Preoperative serum levels of high-sensitivity C-reactive protein (hs-CRP), interleukin-6 (IL-6), albumin (ALB), and complete blood count were assessed for all patients. The parameters were re-evaluated and documented 24, 72, and 120 hours after an operation. Blood sampling was conducted every ±2 hours.

Patients received instructions on using the visual analog scale (VAS) for pain assessment, which ranges from 0 mm to 100 mm. The scoring criteria were defined as follows: 0–4 mm, no pain; 5–44 mm, mild pain; 45–74 mm, moderate pain; and, 75–100 mm, severe pain (19, 20). Upon fully understanding the instructions, the patients were instructed on the effective use of this method to self-assess their pain intensity. The higher dose (40 mg) had a longer analgesic duration (10.6 hours for 40 mg vs. 6.9 hours for 20 mg), and the proportion of patients requiring rescue analgesia within 24 hours was significantly lower in the 40 mg group (66% vs. 81%) (21). We chose 6 hours as the first assessment time point for two practical reasons: it allowed sufficient time for the residual effects of intraoperative sedatives and anesthetics to dissipate, ensuring that patients could provide reliable and consistent pain ratings; and it aligns with our clinical routine for postoperative monitoring, making the findings more applicable to real-world practice. In keeping with our study objectives, we deliberately selected the postoperative time points of 6, 12, 24, 72, and 120 hours for assessment. Based on the pain assessment results, a decision was made to administer rescue analgesia with tramadol injection, and the frequency and total dosage of tramadol were documented. The patients were observed for postoperative complications, including hematemesis, melena, perforation, and infection, in addition to adverse reactions, such as nausea, vomiting, and drowsiness. All occurring events were addressed promptly.

### 2.7 Statistical analysis

Data were analyzed using SPSS software Version 29.0. Continuous data conforming to a normal distribution were expressed as mean ± standard deviation and compared between groups with the independent samples t-test. Categorical variables were expressed as numbers and percentages, and comparisons were conducted using the chi-square test or Fisher’s exact test. Data that did not conform to a normal distribution were reported as medians with interquartile ranges and were analyzed using the Mann–Whitney U test. All tests were two-sided, and a P-value of less than 0.05 was considered statistically significant.

## 3 Results

### 3.1 Patient enrollment

**Figure 2:**
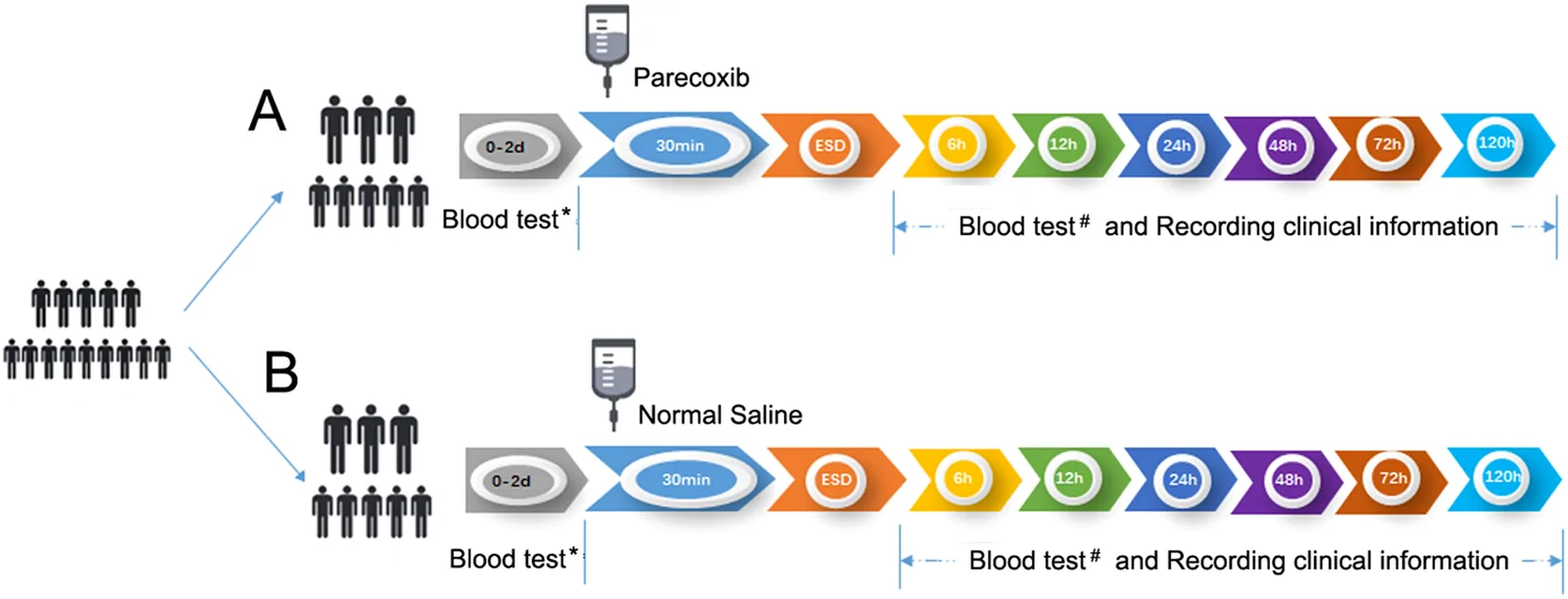
Flowchart of patient enrollment. **(A)** Parecoxib group: intravenous parecoxib sodium 30 min before ESD; blood tests and clinical data recorded preoperatively (0–2 days) and postoperatively. **(B)** Control group: intravenous normal saline 30 min before ESD; blood tests and clinical data recorded preoperatively (0–2 days) and postoperatively.

A total of 93 patients were initially enrolled on the basis of predefined screening criteria. Five patients were excluded because lesion resection involved ≥3/4 of the circumference, which required intraoperative or postoperative corticosteroid administration for stricture prevention. Six additional patients were excluded due to concurrent treatment for gastric polyps or Barrett’s esophagus. The remaining 82 patients were randomly assigned to two groups. Two patients from each group were excluded because of refusal to cooperate with postoperative assessments. In total, 78 patients were included in the final analysis: 40 in the parecoxib group (group A) and 38 in the control group (group B, **Figure 2**).

### 3.2 Comparison of clinicopathological characteristics and preoperative nutritional indices

**Figure 3:**
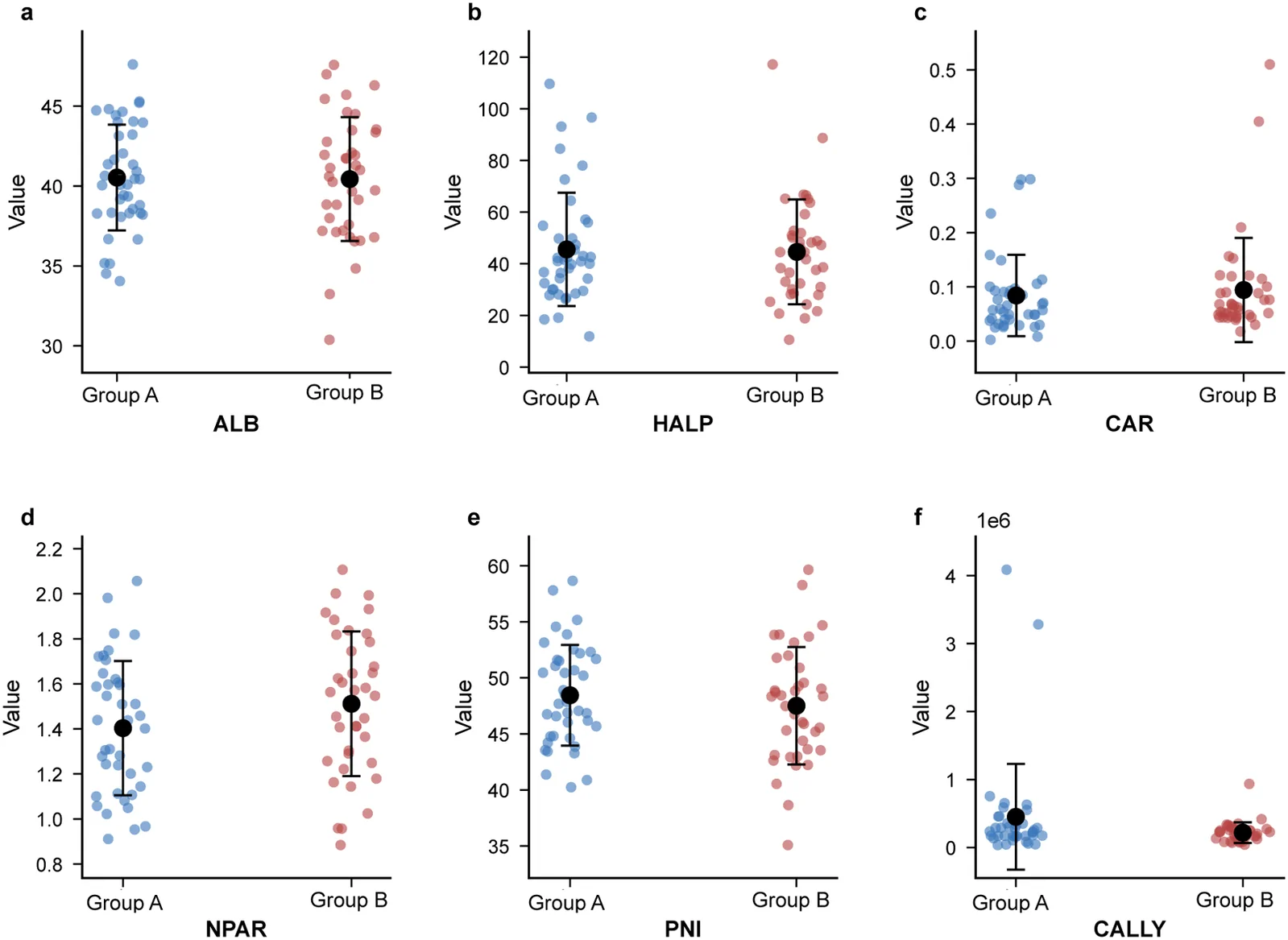
Comparison of preoperative inflammation-related indexes between the two groups. Panels are arranged from left to right and from top to bottom as follows: **(a)** ALB; **(b)** HALP; **(c)** CAR; **(d)** NPAR; **(e)** PNI; **(f)** CALLY. ALB, albumin; HALP, hemoglobin albumin lymphocyte and platelet; NPAR, neutrophil percentage to albumin ratio; CALLY, C-reactive protein and albumin to lymphocyte ratio; PNI, prognostic nutritional index; CAR, C-reactive protein to albumin ratio. No statistically significant differences were observed between the two groups for all indices (all P > 0.05).

The two groups were similar regarding sex, age, height, and weight, with no statistically significant differences (*P* > 0.05). The personal and past medical histories, including smoking, alcohol consumption, hypertension, coronary heart disease, diabetes mellitus, and previous surgeries, were comparable between the groups, indicating no significant differences (**Table 1**, *P* > 0.05). Furthermore, preoperative inflammatory and nutritional indices, including ALB, hemoglobin albumin lymphocyte and platelet (HALP), normalized platelet-albumin ratio (NPAR), C-reactive albumin lymphocyte (CALLY), C-reactive protein to albumin ratio (CAR), and prognostic nutritional index (PNI), were similar across the groups, showing no statistically significant differences (**Figure 3**; **Supplementary Table 1**, *P* > 0.05).

**Table 1:** Summary of clinicopathological and surgical information between the two groups of patients.

Item	Group A (n=40)	Group B (n=38)	χ^2^/t	P
Gender				
Male	22	28	2.956	0.086
Female	18	10		
Age (years)	50-79 (65.2 ± 7.38)	56-83 (64.66 ± 7.97)	-0.312	0.756
Height (cm)	163.38 ± 6.66	164.05 ± 9.88	0.353	0.725
Weight (kg)	61.44 ± 9.46	63.26 ± 10.70	0.799	0.427
Smoking history				
Yes	24	25	0.28	0.597
No	16	13		
Drinking history				
Yes	22	22	0.971	0.324
No	18	16		
Hypertension				
Yes	31	29	0.015	0.901
No	9	9		
Coronary heart disease				
Yes	3	3	0.004	0.948
No	37	35		
Diabetes mellitus				
Yes	0	4	2.538	0.111
No	40	34		
History of surgery				
Yes	23	23	0.074	0.786
No	17	15		

group A: the parecoxib sodium group; group B: the control group; cm: centimeter; kg: kilogram.

### 3.3 Comparison of operative and postoperative pathological findings

The surgical outcomes and postoperative pathological characteristics were comparable between the two groups. No significant differences were observed in lesion location, maximum lesion diameter, resection area, depth of invasion, pathological type, duration of surgery, or duration of anesthesia (**Table 2**, *P* > 0.05).

**Table 2:** Summary of operative information for the two patient groups.

Item	Group A (n=40)	Group B (n=38)	Statistical value	P
Tumor location				
Upper third	1	4	0.594	0.116
Middle third	27	23		
Lower third	4	8		
Multiple sites	8	3		
Depth of invasion				
M1	6	11	11.56	0.073
M2	22	26		
M3	8	0		
SM	4	1		
Maximum Diameter of Resected Specimen (cm)	3.90 ± 1.17	3.89 ± 1.92	-0.015	0.988
Tumor Size (cm)	2.01 ± 1.15	1.84 ± 1.34	-0.613	0.541
Area of Resected Specimen (cm²)	7.01 ± 3.78	7.11 ± 4.29	0.117	0.907
Histological type				
Low-grade dysplasia	0	1	3.208	0.361
High-grade dysplasia	6	10		
Squamous cell carcinoma	30	25		
Mixed	1	2		
Adenoid cystic carcinoma	1	0		
Anesthesia Time (minutes)	75.0 ± 24.86	78.82 ± 33.92	0.569	0.571
Operative Time (minutes)	56.5 ± 24.08	60.53 ± 33.28	0.614	0.541

group A: the parecoxib sodium group; group B: the control group.

### 3.4 Comparison of postoperative pain and analgesic use

**Figure 4:**
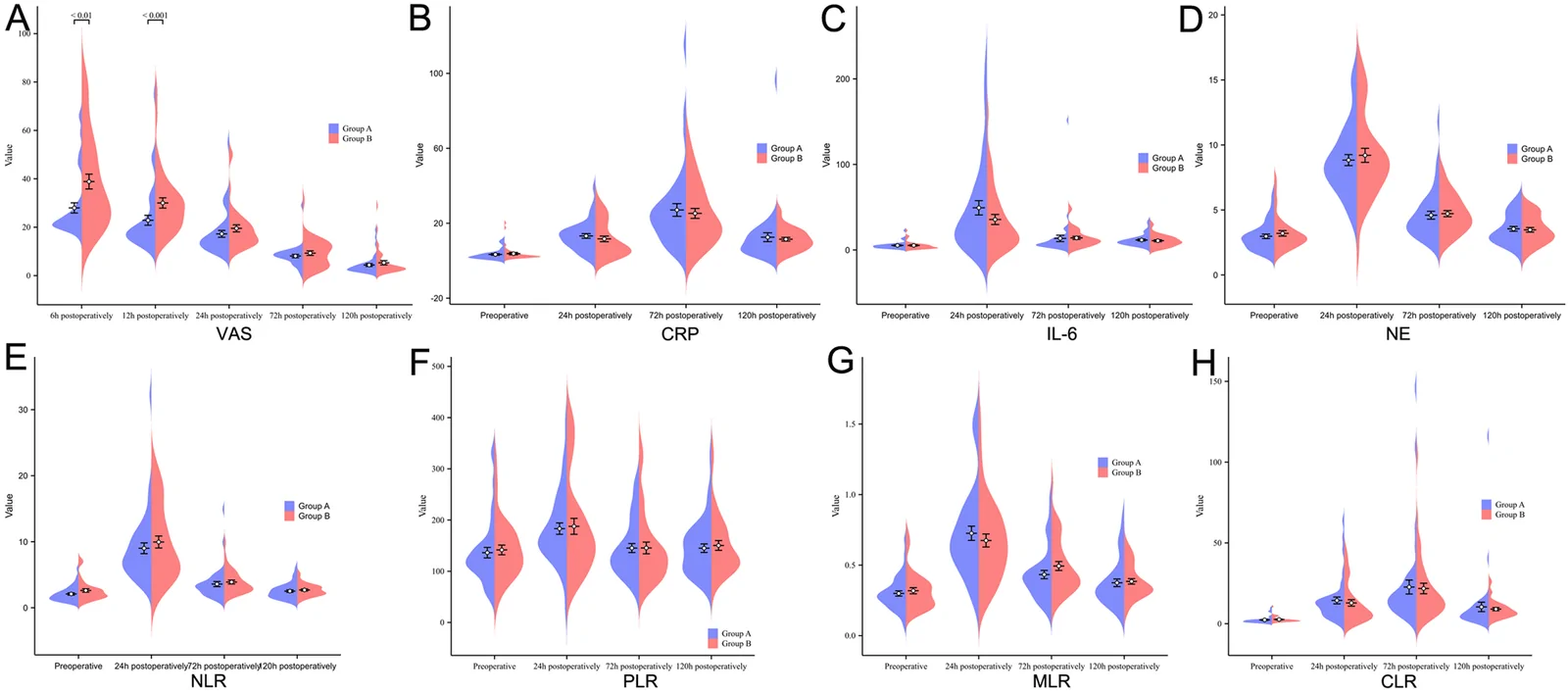
Comparison of postoperative VAS scores and inflammatory marker levels between the two groups. **(A)** VAS scores at 6, 12, 24, 72, and 120 hours postoperatively; **(B)** NE; **(C)** CRP; **(D)** NLR; **(E)** PLR; **(F)** MLR; **(G)** CLR; **(H)** IL-6. VAS, visual analog scale; NE, neutrophil; CRP, C-reactive protein; NLR, neutrophil to lymphocyte ratio; PLR, platelet to lymphocyte ratio; MLR, monocyte to lymphocyte ratio; CLR, C-reactive protein to lymphocyte ratio; IL-6, interleukin-6. At 6 and 12 hours postoperatively, VAS scores were significantly lower in the medication group than in the control group (*P < 0.05). No significant differences were observed for VAS scores at 24, 72, and 120 hours, or for any inflammatory marker at any time point (all P > 0.05).

The comparison of postoperative VAS scores between the groups indicated that pain scores in the medication group were significantly lower than those in the control group at both 6 hours (**Figure 4A**, *P* < 0.05) and 12 hours (*P* < 0.05) postoperatively. VAS scores at 24, 72, and 120 hours were comparable, showing no statistically significant differences (**Table 3**, *P* > 0.05). The frequency and total dosage of postoperative tramadol use were comparable between the groups, indicating no significant difference (**Table 4**, *P* > 0.05).

**Table 3:** Comparison of postoperative VAS Scores between the two groups.

Test time	Group	Number	Minimum	Maximum	Median	Interquartile range	Lower quartile	Upper quartile	Mean	Standard deviation	Standard error	P
6h postoperatively	group A (n=40)	40	16	67	22.500	6.000	20.750	26.750	27.975	13.285	2.101	0.005
	group B (n=38)	38	16	81	31.500	26.500	23.500	50.000	38.868	18.871	3.061	
12h postoperatively	group A (n=40)	40	10	75	19.000	8.750	15.750	24.500	22.850	12.947	2.047	0.017
	group B (n=38)	38	13	76	28.500	13.500	21.000	34.500	30.026	13.095	2.124	
24h postoperatively	group A (n=40)	40	8	55	14.500	7.500	11.000	18.500	17.250	8.949	1.415	0.267
	group B (n=38)	38	10	50	18.000	7.750	13.250	21.000	19.526	9.031	1.465	
72h postoperatively	group A (n=40)	40	0	29	8.000	3.250	6.750	10.000	8.075	4.323	0.683	0.298
	group B (n=38)	38	2	31	9.500	8.750	4.000	12.750	9.289	5.831	0.946	
120h postoperatively	group A (n=40)	40	1	19	3.000	3.000	2.000	5.000	4.375	3.787	0.599	0.378
	group B (n=38)	38	1	29	3.000	3.500	2.250	5.750	5.316	5.463	0.886	

group A: the parecoxib sodium group; group B: the control group.

**Table 4:** Postoperative pain and medication use in the two groups.

Category	Group A	Group B	χ^2^/t	P
Frequency of analgesic administration (n)				
Within 0–6 hours	6	10	2.035	0.844
Within 6–12 hours	4	4		
Within 12–24 hours	2	1		
Within 24–48 hours	4	3		
Within 48–72 hours	2	1		
Within 72–120 hours	3	2		
Total supplemental analgesic dose (mg)				
Within 0–6 hours	600	1000	0	1
Within 6–12 hours	400	400		
Within 12–24 hours	200	100		
Within 24–48 hours	400	400		
Within 48–72 hours	300	100		
Within 72–120 hours	300	200		

group A: the parecoxib sodium group; group B: the control group; VAS, visual analog scale.

### 3.5 Comparison of inflammatory marker levels

Preoperative inflammatory markers, including neutrophil (NE), CRP, neutrophil to lymphocyte ratio (NLR), platelet to lymphocyte ratio (PLR), monocyte to lymphocyte ratio (MLR), C-reactive protein to lymphocyte ratio (CLR), and IL-6, were comparable across the groups (**Supplementary Table 2**). Analysis of inflammatory markers at 24, 72, and 120 hours postoperatively indicated that values in the medication group were generally lower than those in the control group; however, the differences were nonsignificant (**Figures 4B–H**; **Supplementary Table 2**, *P* > 0.05).

### 3.6 Comparison of postoperative adverse reactions

Postoperative adverse reactions comprised retrosternal pain, pharyngeal pain, abdominal pain, abdominal distension, nausea, vomiting, cough, sputum production, fever, dizziness, hoarseness, dysphagia, acid reflux, and belching. The incidence rates of these adverse reactions were comparable between the two groups, with no statistically significant differences (**Table 5**, all *P* > 0.05).

**Table 5:** Comparison of postoperative adverse reactions and symptoms between the two groups.

Adverse reactions/symptoms	Group A (n=40)	Group B (n=38)	Statistical value	P
Chest pain			1.052	0.305
Yes	24	27		
No	16	11		
Pharyngeal pain			0.007	0.935
Yes	4	5		
No	36	33		
Abdominal pain			1.344	0.246
Yes	8	4		
No	32	34		
Abdominal distension			0.001	0.971
Yes	1	1		
No	39	37		
Nausea			0.969	0.325
Yes	1	4		
No	39	34		
Vomiting			0.321	0.571
Yes	1	3		
No	39	35		
Cough			0.004	0.953
Yes	2	3		
No	38	35		
Sputum production			0.002	0.964
Yes	1	2		
No	39	36		
Fever			0.746	0.388
Yes	2	5		
No	38	33		
Dizziness			0.004	0.953
Yes	2	3		
No	38	35		
Hoarseness			0.001	0.971
Yes	1	1		
No	39	37		
Dysphagia			0.962	0.327
Yes	1	0		
No	39	38		
Acid Reflux			0.001	0.979
Yes	0	1		
No	40	37		
Belching			0.001	0.979
Yes	0	1		
No	40	37		

group A: the parecoxib sodium group; group B: the control group.

## 4 Discussion

Pain is defined as an unpleasant sensory and emotional experience associated with actual or potential tissue damage. It functions as an essential self-protection mechanism and a basic physiological characteristic (22). Inadequately managed acute postoperative pain can elicit stress responses, leading to cardiovascular complications, including tachycardia, hypertension, and elevated myocardial oxygen consumption. It may also lead to respiratory depression, endocrine dysfunction, and various systemic disturbances. Moreover, inadequate pain management is linked to prolonged wound healing and the risk of chronic pain development (23).

Postoperative pain frequently occurs in patients who have undergone ESD. Multiple strategies have been investigated to effectively mitigate this pain. Jung JH et al. (24) reported that the application of topical lidocaine significantly alleviated post-ESD pain while not markedly increasing adverse reactions. Choi HS and colleagues (3) similarly found that transdermal fentanyl patches offered comparable analgesic effects. Lee HW et al. (25) demonstrated that patients receiving dexamethasone after ESD experienced significantly lower pain levels and overall pain scores than those receiving a placebo. However, these interventions were implemented after the onset of pain, that is, the patients had already experienced pain, rather than preventing its occurrence. Moreover, these studies primarily focused on subjective pain assessments and did not include objective assessment of related inflammatory biomarkers. Furthermore, most current studies concentrate on pain management following gastric or colorectal ESD, with limited evidence pertaining specifically to esophageal ESD. Given the anatomical and neurophysiological distinctions between the esophagus and other gastrointestinal segments, further targeted research in this field is necessary.

Preemptive analgesia, defined as the administration of analgesic interventions before the onset of noxious stimuli, seeks to prevent central sensitization and mitigate subsequent inflammatory and incisional injury. This method has demonstrated greater efficacy than similar interventions performed after surgery (26). A prospective randomized trial (15) confirmed that parecoxib sodium effectively alleviates pain and reduces the demand for opioids after transarterial chemoembolization for hepatocellular carcinoma, demonstrating a favorable safety profile, good tolerability, and enhanced overall patient comfort. Research conducted by the Chiang Mai University Faculty of Medicine (27) demonstrated that the prophylactic administration of parecoxib sodium before thoracotomy considerably reduces early postoperative pain and improves the quality of analgesia. Furthermore, a multiple-criteria decision analysis (28) identified that among the commonly used analgesics in gastrointestinal surgery (including acetaminophen, diclofenac, ketorolac, metamizole, morphine, nefopam, parecoxib, and tramadol), parecoxib exhibited the most favorable efficacy and safety profile. Numerous studies (29–35) have shown that the preoperative prophylactic use of parecoxib sodium, owing to its anti-inflammatory, analgesic, and antioxidant properties, can mitigate postoperative anxiety and pain, enhance sedation-related dysfunction, and lower the incidence of adverse reactions such as delirium, especially in older patients.

Esophageal ESD results in a mucosal defect, potentially causing postoperative symptoms, including retrosternal pain, odynophagia, and pharyngeal discomfort. The implementation of preemptive analgesia is crucial for effectively managing pain following ESD. This study assessed pain levels with VAS scores at five time points: 6, 12, 24, 72, and 120 hours after the procedure. The experimental group exhibited significantly lower pain scores than the control group at 6 and 12 hours postoperatively (*P* < 0.05). Statistical analysis revealed no significant differences between the groups at 24, 72, and 120 hours (*P* > 0.05). The findings suggest that administering parecoxib sodium 30 minutes before ESD effectively reduces early postoperative pain; however, it does not considerably affect pain in later phases.

Opioids are widely used for postoperative analgesia, but their clinical effectiveness is constrained by various adverse effects, including hypotension, bradycardia, respiratory depression, nausea, vomiting, shivering, and pruritus (36). This study utilized intramuscular tramadol as a rescue analgesic for patients with severe postoperative pain. The experimental group exhibited a reduced need for analgesia within the initial 6 hours. However, the overall frequency and total dosage of tramadol administered were comparable between the groups. In the context of safety assessment, postoperative follow-up indicated that mild adverse reactions, including nausea, vomiting, fever, and dizziness, were observed in both groups. Nevertheless, the incidence rates of these events did not significantly vary between the groups (*P* > 0.05), and all cases were resolved with appropriate management. These results confirm that preemptive analgesia with parecoxib sodium before ESD for early esophageal cancer not only provides exceptional short-term analgesic efficacy but also demonstrates a favorable safety profile.

Parecoxib sodium, which is a highly selective cyclooxygenase-2 inhibitor, demonstrates superior gastrointestinal safety profile to conventional nonsteroidal anti-inflammatory drugs. The inflammatory response initiated by tissue injury is a key factor in the pain experienced after esophageal ESD. In ESD, high-frequency electrosurgery for cutting and coagulation results in localized tissue damage, which subsequently activates the inflammatory cascade. Thus, managing this localized inflammatory response may be an effective strategy for reducing postoperative pain. IL-6 is a crucial pro-inflammatory cytokine involved in an acute-phase response, increasing rapidly in response to stress and demonstrating notable pain-sensitizing effects; its concentration indicates the extent of tissue injury (37). CRP is a nonspecific acute-phase protein produced by the liver that activates the complement system and enhances phagocytic function. Its levels considerably increase in response to tissue damage (38). CRP and IL-6 levels serve as important serum markers of the inflammatory process, and their fluctuations can be used in the assessment of a systemic inflammatory response’s status.

In this study, preoperative inflammatory and nutritional indices, such as ALB, HALP, NPAR, CALLY, CAR, and PNI, were comparable between the groups, indicating balanced baseline characteristics. Postoperatively, inflammatory markers, such as NE, NLR, PLR, MLR, and IL-6, reached their peak at 24 hours and subsequently declined in both groups. The differences did not reach statistical significance, but the values for these markers were consistently lower in the medication group than in the control group. CRP levels peaked at 72 hours postoperatively and were lower in the experimental group, similar to the other markers. However, this difference did not reach statistical significance. The observed trend may be attributed to the dual mechanism of action of parecoxib sodium. First, it modulates the excitability of primary sensory neurons, reducing their efficiency in transmitting nociceptive signals and thereby mediating its analgesic effect. Second, by inhibiting COX-2 expression and modulating the arachidonic acid metabolic pathway, it reduces prostaglandin synthesis. The anti-inflammatory effect is likely a fundamental mechanism underlying the observed analgesia.

Given that gastroesophageal reflux and PPI use may affect post-ESD chest pain, we re-examined the baseline data and found that the distributions of reflux esophagitis (Los Angeles grade B: 2 cases in the Group B and 2 in the Group A), Barrett’s esophagus (diagnosed by NBI endoscopy, lesion length <1cm: 2 cases in Group B and 1 in Group A), and preoperative irregular PPI use (3 cases in Group B and 2 in Group A) were highly balanced between the two groups. All patients uniformly discontinued oral PPIs on the day of surgery and continued to withhold them for 48 hours postoperatively according to a standardized protocol, resulting in identical perioperative PPI exposure. Postoperatively, only 2 patients in each group received intravenous PPIs due to subxiphoid or abdominal pain, while the remaining patients with mild discomfort either received no treatment or only chewable hydrotalcite tablets, which do not significantly affect gastric acid secretion. Based on these data, the baseline prevalence of reflux esophagitis, Barrett’s esophagus, and prior PPI use was highly similar between the two groups, and the perioperative PPI management protocol was uniform and consistently applied. Therefore, we believe that these reflux-related factors were evenly distributed between the groups and do not constitute significant confounders that could explain the intergroup differences in postoperative pain scores or analgesic requirements.

Research on post-ESD pain is currently limited, and there is an absence of a standardized assessment framework is lacking. In clinical practice, postoperative pain is frequently characterized by the need for analgesic rescue medication, potentially resulting in underreporting, because not all instances of pain are documented. Additionally, research utilizing various pain scales is vulnerable to cultural and other confounding variables. This research employed the VAS for evaluating pain. The continuous variable nature facilitates precise tracking of dynamic changes in pain intensity, offering a more sensitive quantitative measure for clinical research. Pain is a subjective experience influenced by the patient’s physiological condition, psychological state, personal history, and sociocultural background. Therefore, the establishment of more precise and objective evaluation criteria is an essential requirement.

The significant difference observed between the 6-hour and 12-hour postoperative time points—the only time-point comparison that reached statistical significance—can be attributed to the following reasons. First, although the peak analgesic effect of parecoxib sodium occurs at approximately 2 hours, its clinical analgesic duration can be extended to about 10.6 hours, as previously described in the literature (21). At 6 hours postoperatively, the plasma concentration of valdecoxib (the active metabolite) remains relatively high, providing effective COX-2 inhibition and sustained pain relief. By 12 hours, the drug concentration has declined markedly, resulting in a measurable rebound in pain perception. Second, the inflammatory response in the early postoperative period is dynamic. Pro-inflammatory cytokines (e.g., IL-6, NE) typically peak within 24 hours after surgical trauma, and the analgesic effect of parecoxib partially overlaps with the early phase of drug exposure and inflammation. We hypothesize that at 6 hours, the drug still exerts its anti-inflammatory action; by 12 hours, the decline in drug levels may allow the inflammatory cascade to become clinically more evident, thereby leading to increased pain. Third, the other time points (24, 72, and 120 hours) did not show statistically significant differences. This is plausible because after 24 hours the drug has been largely eliminated, and pain is primarily influenced by tissue healing and individual variability rather than by the pharmacological effect of a single dose. As postoperative pain naturally subsides, the between-group differences diminish, making it difficult to reach statistical significance.

Although parecoxib sodium significantly reduced pain scores at 6 and 12 hours postoperatively, the frequency and total dose of rescue tramadol did not differ significantly between the two groups. Our analysis of the possible reasons is as follows: First, postoperative pain is multidimensional in nature. A reduction in early resting pain does not necessarily equate to a commensurate and significant alleviation of movement-evoked pain (e.g., during coughing or turning over) or visceral traction pain, and our preoperative intervention may have had relatively limited coverage of the latter components. Second, in terms of absolute numbers, the proportions of patients requiring rescue analgesics were similar between the two groups (34.2% vs. 30.0%), with an absolute risk difference of only 4.2%. Finally, the sample size of this study was relatively small, and the power calculation was primarily based on the continuous variable of VAS scores at the design stage; thus, the statistical power for detecting differences in rescue analgesic utilization rates may have been insufficient. Therefore, the conclusion of our study should be objectively framed as follows: this intervention strategy provides superior early analgesia for resting pain without increasing the postoperative requirement for rescue analgesics or the risk of adverse reactions; however, translating this early analgesic advantage into an overall opioid-sparing benefit will require further validation in studies with larger sample sizes.

In the esophageal field, animal studies have shown that the selective COX-2 inhibitor celecoxib can significantly delay healing of rat esophageal ulcers by downregulating the HGF/c-Met–ERK2 signaling pathway, thereby inhibiting ulcer-triggered epithelial cell proliferation (39). Nevertheless, we believe that several factors reduce the potential impact of such risks in our patient population: First, lesion characteristics and procedural integrity. All patients enrolled in our study had early-stage esophageal cancer with lesions confined to the mucosal layer and no invasion of the muscularis propria. During the ESD procedures, we achieved en bloc curative resection in all cases without any injury to the muscularis propria. The healing trajectory of such mucosal-predominant defects differs from that of deeper surgical wounds, and the preservation of the muscular layer likely provides a more favorable environment for mucosal regeneration. Second, we administered only a single dose of parecoxib sodium for postoperative analgesia. This single, short-term exposure is substantially different from the repeated or prolonged dosing regimens used in the animal studies that demonstrated delayed ulcer healing. Given the limited temporal presence of the drug’s pharmacokinetic effects, we consider that the window of COX-2 inhibition in our patients is too narrow to exert a clinically meaningful impact on the mucosal healing process after ESD, which typically spans several weeks. Third, the favorable gastrointestinal safety profile of parecoxib sodium. In a 7-day endoscopic trial conducted in healthy subjects, none of the participants receiving parecoxib developed gastroduodenal ulcers (40). Although these data do not directly address the issue of ulcer healing in the presence of an existing mucosal defect, they do support the overall gastrointestinal safety of short-term parecoxib use. For these reasons, we consider that the potential risk of delayed ulcer healing due to COX-2 inhibition in this study is minimal and did not affect our clinical outcomes.

This study represents the first application of preemptive analgesia in esophageal ESD and demonstrates the efficacy and safety of a preemptive parecoxib sodium approach for pain management after ESD in patients with early esophageal cancer and mucosal defects covering less than three-quarters of the esophageal circumference. The analgesic effect may be associated with its anti-inflammatory properties. This study has several limitations. First, the enrolled patients were generally in good health and lacked significant comorbidities. Thus, the findings may not be applicable to individuals with poor systemic conditions or severe complications. Second, the study design employed a blank control (normal saline) and excluded active comparators with other analgesic agents. The study examined only one intravenous dosing regimen, thereby leaving the comparison of efficacy and safety between single and multiple doses unaddressed. Third, patients with postoperative mucosal defects involving more than three-quarters of the esophageal circumference were not enrolled in this study because they required glucocorticoid administration during or after the procedure to prevent stricture, which could interfere with inflammatory markers. Whether the preemptive analgesia strategy with parecoxib sodium is also feasible in this patient population warrants further investigation. In conclusion, this single-center study, characterized by a relatively small sample size, necessitates future large-scale, multicenter prospective studies to validate these findings and contribute to the establishment of a standardized pain assessment system.

## 5 Conclusion

Preemptive analgesia with parecoxib sodium can alleviate early postoperative pain in patients with early esophageal cancer and precancerous lesions who undergo ESD for mucosal defects involving less than three-quarters of the esophageal circumference, without increasing adverse reactions or the subsequent use of analgesic drugs.

## Data Availability

The datasets presented in this study can be found in online repositories. The names of the repository/repositories and accession number(s) can be found in the article/**Supplementary Material**.

## References

[1] Iwai N Dohi O Yamada S Harusato A Horii R Yagi N . Prognostic risk factors associated with esophageal squamous cell carcinoma patients undergoing endoscopic submucosal dissection: a multi-center cohort study. Surg Endosc. (2022) 36:2279–89. doi: 10.1007/s00464-021-08502-1 33860352

[2] Duan Y Jia W Liang Y Zhang H Liu L Li Y . Progress in the treatment and prevention of esophageal stenosis after endoscopic submucosal dissection. Clin Res Hepatol Gastroenterol. (2024) 48:102290. doi: 10.1016/j.clinre.2024.102290 38311060

[3] Choi HS Kim KO Chun HJ Keum B Seo YS Kim YS . The efficacy of transdermal fentanyl for pain relief after endoscopic submucosal dissection: a prospective, randomised controlled trial. Dig Liver Dis. (2012) 44:925–29. doi: 10.1016/j.dld.2012.06.015 22824834

[4] Nishizawa T Yahagi N . Endoscopic mucosal resection and endoscopic submucosal dissection: technique and new directions. Curr Opin Gastroenterol. (2017) 33:315–19. doi: 10.1097/MOG.0000000000000388 28704212

[5] Kim SY Jung SW Choe JW Jung YK Yim HJ Seo YS . Predictive factors for pain after endoscopic resection of gastric tumors. Dig Dis Sci. (2016) 61:3560–64. doi: 10.1007/s10620-016-4325-9 27696098

[6] Jung DH Youn YH Kim JH Park H . Factors influencing development of pain after gastric endoscopic submucosal dissection: a randomized controlled trial. Endoscopy. (2015) 47:1119–23. doi: 10.1055/s-0034-1391234 26165736

[7] Sakai M Sohda M Saito H Ubukata Y Nakazawa N Kuriyama K . Chest pain after endoscopic submucosal dissection for esophageal cancer: the simple and clinically useful surrogate marker for transmural thermal injury by electrocoagulation. Digestion. (2021) 102:607–14. doi: 10.1159/000514743 33032290

[8] Onogi F Araki H Ibuka T Seko Y Nishiwaki S Yamada T . Transmural air leak: a computed tomographic finding following endoscopic submucosal dissection of gastric tumors. Endoscopy. (2010) 42:441–47. doi: 10.1055/s-0030-1250500 20432207

[9] Lee H Cheoi KS Chung H Park JC Shin SK Lee SK . Clinical features and predictive factors of coagulation syndrome after endoscopic submucosal dissection for early gastric neoplasm. Gastric Cancer. (2012) 15:83–90. doi: 10.1007/s10120-011-0073-x 21761134

[10] Probst A Maerkl B Bittinger M Messmann H . Gastric ischemia following endoscopic submucosal dissection of early gastric cancer. Gastric Cancer. (2010) 13:58–61. doi: 10.1007/s10120-009-0539-2 20373077

[11] Parkman HP Jones MP . Tests of gastric neuromuscular function. Gastroenterology. (2009) 136:1526–43. doi: 10.1053/j.gastro.2009.02.072 19293005

[12] Ochi M Yamamoto A Suematsu S Okahisa T Tanaka S Nakai Y . High Joule heat as a risk factor for post-endoscopic submucosal dissection electrocoagulation syndrome: a multicenter prospective study. World J Gastrointest Endosc. (2024) 16:668–77. doi: 10.4253/wjge.v16.i12.668 39735389 PMC11669959

[13] Ochi M Kawagoe R Kamoshida T Ohkawara A Ohkawara H Kato J . High total Joule heat increases the risk of post-endoscopic submucosal dissection electrocoagulation syndrome after colorectal endoscopic submucosal dissection. World J Gastroenterol. (2021) 27:6442–52. doi: 10.3748/wjg.v27.i38.6442 34720533 PMC8517781

[14] Dos SV Groppo FC de Toledo J Crivelini MM Neto JR de Oliveira EC . Effect of parecoxib and dexamethasone on the temporomandibular joint of orchiectomized rats: morphological and immunological analysis. Arch Oral Biol. (2025) 179:106381. doi: 10.1016/j.archoralbio.2025.106381 40850211

[15] Lv N Kong Y Mu L Pan T Xie Q Zhao M . Effect of perioperative parecoxib sodium on postoperative pain control for transcatheter arterial chemoembolization for inoperable hepatocellular carcinoma: a prospective randomized trial. Eur Radiol. (2016) 26:3492–99. doi: 10.1007/s00330-016-4207-8 26801163

[16] Nakatani S Hayasaka J Ishii T Kikuchi D Ochiai Y Nomura K . Factors influencing resection time in endoscopic submucosal dissection for rectal neuroendocrine tumors. Den Open. (2026) 6:e70199. doi: 10.1002/deo2.70199 40909210 PMC12404866

[17] Xing J Li P . Diagnosis and treatment of early esophageal squamous cell carcinoma and precancerous lesions. Zhonghua Nei Ke Za Zhi. (2020) 59:318–21. doi: 10.3760/cma.j.cn112138-20200210-00061 32209201

[18] Yuan ML Zeng XS Yu XC Liu FZ Wang GQ Wei WQ . Analysis on the effect of iodine staining endoscopy in screening for early esophageal cancer. Zhonghua Yi Xue Za Zhi. (2025) 105:1515–19. doi: 10.3760/cma.j.cn112137-20241009-02279 40374336

[19] Hawker GA Mian S Kendzerska T French M . Measures of adult pain: visual analog scale for pain (VAS Pain), numeric rating scale for pain (NRS Pain), McGill pain questionnaire (MPQ), short-form McGill pain questionnaire (SF-MPQ), chronic pain grade scale (CPGS), short form-36 bodily pain scale (SF-36 BPS), and measure of intermittent and constant osteoarthritis pain (ICOAP). Arthritis Care Res (Hoboken). (2011) 63 Suppl 11:S240–52. doi: 10.1002/acr.20543 22588748

[20] Wan L Zhao Q Chen J Fan BF Gao CR Hu L . Expert consensus on the application of pain evaluation questionnaires in China (2020 edition). Chin J Painology. (2020) 16:177–87. doi: 10.3760/cma.j.cn101379-20190915-00075 30704229

[21] Lloyd R Derry S Moore RA McQuay HJ . Intravenous or intramuscular parecoxib for acute postoperative pain in adults. Cochrane Database Systematic Rev. (2009) 2009:CD004771. doi: 10.1002/14651858.CD004771.pub4 19370610 PMC6540719

[22] Aweke Z Seyoum F Shitemaw T Dulla S . Comparison of preemptive paracetamol, paracetamol-diclofenac & paracetamol-tramadol combination on postoperative pain after elective abdominal surgery under general anesthesia, Ethiopia: a randomized control trial study, 2018. BMC Anesthesiol. (2020) 20:191. doi: 10.1186/s12871-020-01115-6 32753063 PMC7401211

[23] Liu L Zhao G Dou Y Zhang H Li J Wang X . Analgesic effects of perioperative acupuncture methods: a narrative review. Med (Baltimore). (2023) 102:e35759. doi: 10.1097/MD.0000000000035759 37904453 PMC10615492

[24] Jung JH Jang HJ Bang CS Park GD Shin WG Kim KH . Efficacy of submucosal bupivacaine injection for pain relief after endoscopic submucosal dissection: a multicenter, prospective, randomized controlled, and double-blind trial. Med (Baltimore). (2019) 98:e15360. doi: 10.1097/MD.0000000000015360 31027120 PMC6831157

[25] Lee HW Lee H Chung H Park JC Shin SK Lee SK . The efficacy of single-dose postoperative intravenous dexamethasone for pain relief after endoscopic submucosal dissection for gastric neoplasm. Surg Endosc. (2014) 28:2334–41. doi: 10.1007/s00464-014-3463-4 24570015

[26] Hu JC Xiong FJ Zhao W Chen HB Yang SM . Effect of oral preemptive analgesia on pain management after total knee arthroplasty: a systematic review and meta-analysis of randomized control trials. Med (Baltimore). (2025) 104:e44518. doi: 10.1097/MD.0000000000044518 40958242 PMC12440553

[27] Pipanmekaporn T Punjasawadwong Y Charuluxananan S Lapisatepun W Charuluxananan S Saeteng S . The effectiveness of intravenous parecoxib on the incidence of ipsilateral shoulder pain after thoracotomy: a randomized, double-blind, placebo-controlled trial. J Cardiothorac Vasc Anesth. (2018) 32:302–08. doi: 10.1053/j.jvca.2017.05.048 29223722

[28] Schug S Pogatzki-Zahn E Phillips LD Doleman B Barden J Kranke P . Multi-criteria decision analysis to develop an efficacy-safety profile of parenteral analgesics used in the treatment of postoperative pain. J Pain Res. (2020) 13:1969–77. doi: 10.2147/JPR.S255921 32801852 PMC7415456

[29] Tan Q Li Y Zhou Q . Prophylactic administration of parecoxib sodium for postoperative delirium in hip surgery of the elderly: a prospective, randomized, placebo-controlled trial. Rev Assoc Med Bras (1992). (2025) 71:e20250548. doi: 10.1590/1806-9282.20250548 40990756 PMC12452156

[30] Wang JH Liu T Bai Y Wang Y Chen L . The effect of parecoxib sodium on postoperative delirium in elderly patients with hip arthroplasty. Front Pharmacol. (2023) 14:947982. doi: 10.3389/fphar.2023.947982 37025488 PMC10072322

[31] Zhang F Wang C Li K Chen B Li Y . Effect of parecoxib and transversus abdominis plane block on postoperative anxiety and cognition: a randomized controlled-trial. J Pak Med Assoc. (2025) 75:877–82. doi: 10.47391/JPMA.20349 40698461

[32] Daojun Z Yuling T Yingzhe X Meiling D Yuping L Kun L . Effect of parecoxib on postoperative delirium in patients with hyperlipidemia: a randomized, double-blind, single-center, superiority trial. Int J Surg. (2025) 111:2903–13. doi: 10.1097/JS9.0000000000002286 39903567 PMC12175806

[33] Sarridou D Gkiouliava A Argiriadou H Anastasiou A Kesisoglou I Vretzakis G . Haemodynamics, side effects and safety of the combination of continuous femoral nerve block and intravenous parecoxib for pain management after total knee arthroplasty: a pilot study. Orthop Rev (Pavia). (2024) 16:122536. doi: 10.52965/001c.122536 39286466 PMC11405027

[34] Li Y Peng Y . Effect of parecoxib on postoperative cognitive function and analgesic safety in elderly patients undergoing gastrointestinal tumor resection: a retrospective study. Biomol BioMed. (2025) 25:720–26. doi: 10.17305/bb.2024.11042 PMC12010987 39284279

[35] Yin WY Peng T Guo BC Yang J Zhang X Li Y . Effects of parecoxib on postoperative cognitive dysfunction and serum levels of NSE and S100beta in elderly patients undergoing surgery. Eur Rev Med Pharmacol Sci. (2024) 28:278–87. doi: 10.26355/eurrev_202401_34914 38235879

[36] Tadesse MA Alemu EA Allene MD Ayele AY Molla M . Efficacy and safety of midazolam compared to fentanyl as adjuvants to hyperbaric bupivacaine in spinal anesthesia: a systematic review and meta-analysis of randomized controlled trials. BMC Anesthesiol. (2025) 25:397. doi: 10.1186/s12871-025-03261-1 40775683 PMC12330165

[37] Yang X Ma J Li K Chen L Wang Y Zhao G . A comparison of effects of scalp nerve block and local anesthetic infiltration on inflammatory response, hemodynamic response, and postoperative pain in patients undergoing craniotomy for cerebral aneurysms: a randomized controlled trial. BMC Anesthesiol. (2019) 19:91. doi: 10.1186/s12871-019-0760-4 31153358 PMC6545200

[38] Levinson T Wasserman A . C-reactive protein velocity (CRPv) as a new biomarker for the early detection of acute infection/inflammation. Int J Mol Sci. (2022) 23:8100. doi: 10.3390/ijms23158234 35897672 PMC9330915

[39] Baatar D Jones MK Pai R Kawanaka H Szabo IL Moon WS . Selective cyclooxygenase-2 blocker delays healing of esophageal ulcers in rats and inhibits ulceration-triggered c-Met/hepatocyte growth factor receptor induction and extracellular signal-regulated kinase 2 activation. Am J Pathol. (2002) 160:963–72. doi: 10.1016/S0002-9440(10)64918-8 11891194 PMC1867196

[40] Harris SI Stoltz RR LeComte D Hubbard RC . Parecoxib sodium demonstrates gastrointestinal safety comparable to placebo in healthy subjects. J Clin Gastroenterol. (2004) 38:575–80. doi: 10.1097/00004836-200408000-00007 15232360

