# Supplementary material for: Preemptive analgesia with parecoxib sodium alleviates short-term pain after endoscopic submucosal dissection for early esophageal cancer and precancerous lesions: a single-center, double-arm, and randomized controlled study
Source: Front Oncol. 2026 Aug 14;16:1858955. doi: 10.3389/fonc.2026.1858955 (PMC13522748; doi:10.3389/fonc.2026.1858955)
Supplement: Supplementary file 1 [file Table1.docx]

| Table S1. Comparison of preoperative inflammation-related indexes between the two groups | | | | | | | | | | | | |
| --- | --- | --- | --- | --- | --- | --- | --- | --- | --- | --- | --- | --- |
| Test Item | group | Minimum | Maximum | Median | Interquartile Range | Lower Quartile | Upper Quartile | Mean | Standard Deviation | Standard Error | P |  |
| ALB | group A  (n=40) | 34.05 | 47.61 | 40.26 | 5.1 | 38.308 | 43.408 | 40.53 | 3.309 | 0.523 | 0.907 |  |
|  | group B  (n=38) | 30.38 | 47.58 | 40.8 | 5.91 | 37.303 | 43.213 | 40.434 | 3.874 | 0.628 |  |  |
| HALP | group A  (n=40) | 11.878 | 109.653 | 40.958 | 20.953 | 30.085 | 51.038 | 45.536 | 21.921 | 3.466 | 0.843 |  |
|  | group B  (n=38) | 10.573 | 117.164 | 43.928 | 21.349 | 30.315 | 51.664 | 44.584 | 20.29 | 3.292 |  |  |
| NPAR | group A  (n=40) | 0.911 | 2.057 | 1.42 | 0.474 | 1.136 | 1.61 | 1.403 | 0.298 | 0.047 | 0.13 |  |
|  | group B  (n=38) | 0.884 | 2.107 | 1.555 | 0.51 | 1.265 | 1.776 | 1.511 | 0.321 | 0.052 |  |  |
| CALLY | group A  (n=40) | 35417.787 | 4087360 | 245410.3 | 285736.54 | 168843.11 | 454579.65 | 450873.55 | 777710.81 | 122966.88 | 0.074 |  |
|  | group B  (n=38) | 40772.418 | 935347.83 | 204856.67 | 141415.73 | 126730.44 | 268146.17 | 218328.91 | 150953.68 | 24487.921 |  |  |
| PNI | group A  (n=40) | 40.24 | 58.66 | 47.77 | 6.823 | 44.8 | 51.623 | 48.448 | 4.497 | 0.711 | 0.397 |  |
|  | group B  (n=38) | 35.08 | 59.65 | 47.915 | 7.003 | 43.563 | 50.565 | 47.509 | 5.236 | 0.849 |  |  |
| CAR | group A  (n=40) | 0.338 | 2.425 | 0.996 | 0.449 | 0.804 | 1.253 | 1.11 | 0.484 | 0.077 | 0.637 |  |
|  | group B  (n=38) | 0.348 | 2.694 | 1.069 | 0.557 | 0.757 | 1.314 | 1.088 | 0.459 | 0.074 |  |  |

Notes: group A: the parecoxib sodium group; group B: the control group; ALB: Albumin; HALP: hemoglobin albumin lymphocyte and platelet; NPAR: neutrophil percentage to albumin ratio; CALLY: C-reactive rrotein and albumin to lymphocyte ratio; PNI: prognostic nutritional index; CAR: C-reactive rrotein to albumin ratio.
