# Supplementary material for: Preemptive analgesia with parecoxib sodium alleviates short-term pain after endoscopic submucosal dissection for early esophageal cancer and precancerous lesions: a single-center, double-arm, and randomized controlled study
Source: Front Oncol. 2026 Aug 14;16:1858955. doi: 10.3389/fonc.2026.1858955 (PMC13522748; doi:10.3389/fonc.2026.1858955)
Supplement: Supplementary file 2 [file Table2.docx]

| Table S2 Comparison of inflammatory indexes between the two groups | | | | | | | | | | | | |
| --- | --- | --- | --- | --- | --- | --- | --- | --- | --- | --- | --- | --- |
|  | Test time | group | Minimum | Maximum | Median | Interquartile Range | Lower Quartile | Upper Quartile | Mean | Standard Deviation | Standard Error | P |
| NE | Preoperative | group A (n=40) | 1.63 | 6.25 | 2.82 | 1.238 | 2.138 | 3.375 | 2.972 | 1.089 | 0.172 | 0.402 |
|  |  |  |  |  |  |  |  |  |  |  |  |  |
|  |  | group B (n=38) | 1.51 | 7.47 | 2.87 | 1.175 | 2.43 | 3.605 | 3.201 | 1.308 | 0.212 |  |
|  |  |  |  |  |  |  |  |  |  |  |  |  |
|  | 24h postoperatively | group A (n=40) | 4.38 | 15.46 | 8.395 | 3.045 | 7.085 | 10.13 | 8.836 | 2.676 | 0.423 | 0.592 |
|  |  |  |  |  |  |  |  |  |  |  |  |  |
|  |  | group B (n=38) | 1.85 | 16.57 | 8.885 | 3.548 | 7.275 | 10.823 | 9.202 | 3.326 | 0.539 |  |
|  |  |  |  |  |  |  |  |  |  |  |  |  |
|  | 72h postoperatively | group A (n=40) | 1.99 | 11.8 | 4.08 | 1.99 | 3.338 | 5.328 | 4.593 | 1.923 | 0.304 | 0.751 |
|  |  |  |  |  |  |  |  |  |  |  |  |  |
|  |  | group B (n=38) | 2.45 | 8.13 | 4.48 | 1.985 | 3.678 | 5.663 | 4.716 | 1.462 | 0.237 |  |
|  |  |  |  |  |  |  |  |  |  |  |  |  |
|  | 120h postoperatively | group A (n=40) | 1.5 | 5.91 | 3.26 | 1.913 | 2.713 | 4.625 | 3.548 | 1.16 | 0.183 | 0.769 |
|  |  |  |  |  |  |  |  |  |  |  |  |  |
|  |  | group B (n=38) | 1.52 | 5.27 | 3.285 | 1.78 | 2.608 | 4.388 | 3.473 | 1.083 | 0.176 |  |
|  |  |  |  |  |  |  |  |  |  |  |  |  |
|  |  |  |  |  |  |  |  |  |  |  |  |  |
| CRP | Preoperative | group A (n=40) | 0.1 | 11.34 | 2.45 | 2.478 | 1.41 | 3.888 | 3.339 | 2.753 | 0.435 | 0.566 |
|  |  |  |  |  |  |  |  |  |  |  |  |  |
|  |  | group B (n=38) | 0.79 | 20.26 | 2.55 | 1.965 | 1.943 | 3.908 | 3.779 | 3.934 | 0.638 |  |
|  |  |  |  |  |  |  |  |  |  |  |  |  |
|  | 24h postoperatively | group A (n=40) | 1.27 | 39.12 | 12.84 | 8.455 | 8.178 | 16.633 | 13.209 | 7.808 | 1.235 | 0.425 |
|  |  |  |  |  |  |  |  |  |  |  |  |  |
|  |  | group B (n=38) | 2.2 | 33 | 8.245 | 15.215 | 3.948 | 19.163 | 11.655 | 9.274 | 1.504 |  |
|  |  |  |  |  |  |  |  |  |  |  |  |  |
|  | 72h postoperatively | group A (n=40) | 2.46 | 115.39 | 23.53 | 16.548 | 14.028 | 30.575 | 27.078 | 21.355 | 3.377 | 0.673 |
|  |  |  |  |  |  |  |  |  |  |  |  |  |
|  |  | group B (n=38) | 2.49 | 66.6 | 19.55 | 21.84 | 14.643 | 36.483 | 25.251 | 16.269 | 2.639 |  |
|  |  |  |  |  |  |  |  |  |  |  |  |  |
|  | 120h postoperatively | group A (n=40) | 1.28 | 96.36 | 10.01 | 10.59 | 4.525 | 15.115 | 12.542 | 14.882 | 2.353 | 0.68 |
|  |  |  |  |  |  |  |  |  |  |  |  |  |
|  |  | group B (n=38) | 1.87 | 27.32 | 10.59 | 9.058 | 6.715 | 15.773 | 11.468 | 6.047 | 0.981 |  |
|  |  |  |  |  |  |  |  |  |  |  |  |  |
|  |  |  |  |  |  |  |  |  |  |  |  |  |
| NLR | Preoperative | group A (n=40) | 0.847 | 5.9 | 1.794 | 1.206 | 1.28 | 2.486 | 2.091 | 1.116 | 0.176 | 0.095 |
|  |  |  |  |  |  |  |  |  |  |  |  |  |
|  |  | group B (n=38) | 0.733 | 7.361 | 2.146 | 1.305 | 1.591 | 2.896 | 2.615 | 1.594 | 0.259 |  |
|  |  |  |  |  |  |  |  |  |  |  |  |  |
|  | 24h postoperatively | group A (n=40) | 3.086 | 32.255 | 7.877 | 4.63 | 6.267 | 10.896 | 9.01 | 5.164 | 0.817 | 0.438 |
|  |  |  |  |  |  |  |  |  |  |  |  |  |
|  |  | group B (n=38) | 1.445 | 21.899 | 8.238 | 7.55 | 5.757 | 13.308 | 9.958 | 5.606 | 0.909 |  |
|  |  |  |  |  |  |  |  |  |  |  |  |  |
|  | 72h postoperatively | group A (n=40) | 0.909 | 14.937 | 3.253 | 1.762 | 2.242 | 4.004 | 3.588 | 2.423 | 0.383 | 0.523 |
|  |  |  |  |  |  |  |  |  |  |  |  |  |
|  |  | group B (n=38) | 1.509 | 9.915 | 3.327 | 2.143 | 2.616 | 4.759 | 3.896 | 1.757 | 0.285 |  |
|  |  |  |  |  |  |  |  |  |  |  |  |  |
|  | 120h postoperatively | group A (n=40) | 0.882 | 6.084 | 2.344 | 1.445 | 1.613 | 3.058 | 2.522 | 1.18 | 0.187 | 0.468 |
|  |  |  |  |  |  |  |  |  |  |  |  |  |
|  |  | group B (n=38) | 1.061 | 5.344 | 2.494 | 1.163 | 2.05 | 3.213 | 2.694 | 0.943 | 0.153 |  |
|  |  |  |  |  |  |  |  |  |  |  |  |  |
|  |  |  |  |  |  |  |  |  |  |  |  |  |
| PLR | Preoperative | group A (n=40) | 45.353 | 337.255 | 120.945 | 46.635 | 101.011 | 147.645 | 136.303 | 64.967 | 10.272 | 0.704 |
|  |  |  |  |  |  |  |  |  |  |  |  |  |
|  |  | group B (n=38) | 33.036 | 322.222 | 135.35 | 71.92 | 104.413 | 176.333 | 141.654 | 58.526 | 9.494 |  |
|  |  |  |  |  |  |  |  |  |  |  |  |  |
|  | 24h postoperatively | group A (n=40) | 26.087 | 392.222 | 164.271 | 76.761 | 139.722 | 216.484 | 183.205 | 70.367 | 11.126 | 0.814 |
|  |  |  |  |  |  |  |  |  |  |  |  |  |
|  |  | group B (n=38) | 66.667 | 411.594 | 160.631 | 92.908 | 125.746 | 218.653 | 187.693 | 95.992 | 15.572 |  |
|  |  |  |  |  |  |  |  |  |  |  |  |  |
|  | 72h postoperatively | group A (n=40) | 58.333 | 272.059 | 130.552 | 67.909 | 104.181 | 172.091 | 145.286 | 55.535 | 8.781 | 0.99 |
|  |  |  |  |  |  |  |  |  |  |  |  |  |
|  |  | group B (n=38) | 53.659 | 346.575 | 132.306 | 67.173 | 97.944 | 165.117 | 145.473 | 70.614 | 11.455 |  |
|  |  |  |  |  |  |  |  |  |  |  |  |  |
|  | 120h postoperatively | group A (n=40) | 67.66 | 325.806 | 144.22 | 75.091 | 100.54 | 175.631 | 145.009 | 52.818 | 8.351 | 0.674 |
|  |  |  |  |  |  |  |  |  |  |  |  |  |
|  |  | group B (n=38) | 60.386 | 340.952 | 132.627 | 67.085 | 113.419 | 180.504 | 150.348 | 58.832 | 9.544 |  |
|  |  |  |  |  |  |  |  |  |  |  |  |  |
|  |  |  |  |  |  |  |  |  |  |  |  |  |
| MLR | Preoperative | group A (n=40) | 0.069 | 0.681 | 0.28 | 0.114 | 0.232 | 0.346 | 0.299 | 0.119 | 0.019 | 0.516 |
|  |  |  |  |  |  |  |  |  |  |  |  |  |
|  |  | group B (n=38) | 0.159 | 0.727 | 0.295 | 0.149 | 0.22 | 0.368 | 0.318 | 0.134 | 0.022 |  |
|  |  |  |  |  |  |  |  |  |  |  |  |  |
|  | 24h postoperatively | group A (n=40) | 0.246 | 1.536 | 0.669 | 0.329 | 0.522 | 0.851 | 0.726 | 0.319 | 0.05 | 0.459 |
|  |  |  |  |  |  |  |  |  |  |  |  |  |
|  |  | group B (n=38) | 0.29 | 1.545 | 0.644 | 0.382 | 0.472 | 0.853 | 0.675 | 0.284 | 0.046 |  |
|  |  |  |  |  |  |  |  |  |  |  |  |  |
|  | 72h postoperatively | group A (n=40) | 0.142 | 0.987 | 0.414 | 0.165 | 0.313 | 0.478 | 0.433 | 0.187 | 0.03 | 0.159 |
|  |  |  |  |  |  |  |  |  |  |  |  |  |
|  |  | group B (n=38) | 0.216 | 1.085 | 0.428 | 0.224 | 0.363 | 0.587 | 0.494 | 0.195 | 0.032 |  |
|  |  |  |  |  |  |  |  |  |  |  |  |  |
|  | 120h postoperatively | group A (n=40) | 0.112 | 0.855 | 0.331 | 0.18 | 0.256 | 0.436 | 0.375 | 0.171 | 0.027 | 0.75 |
|  |  |  |  |  |  |  |  |  |  |  |  |  |
|  |  | group B (n=38) | 0.166 | 0.651 | 0.341 | 0.159 | 0.312 | 0.471 | 0.385 | 0.119 | 0.019 |  |
|  |  |  |  |  |  |  |  |  |  |  |  |  |
|  |  |  |  |  |  |  |  |  |  |  |  |  |
| CLR | Preoperative | group A (n=40) | 0.09 | 9.922 | 1.617 | 1.54 | 0.88 | 2.42 | 2.355 | 2.286 | 0.362 | 0.572 |
|  |  |  |  |  |  |  |  |  |  |  |  |  |
|  |  | group B (n=38) | 0.49 | 10.679 | 2.087 | 1.289 | 1.493 | 2.783 | 2.621 | 1.844 | 0.299 |  |
|  |  |  |  |  |  |  |  |  |  |  |  |  |
|  | 24h postoperatively | group A (n=40) | 0.81 | 63.745 | 12.084 | 9.153 | 6.047 | 15.199 | 14.398 | 13.372 | 2.114 | 0.599 |
|  |  |  |  |  |  |  |  |  |  |  |  |  |
|  |  | group B (n=38) | 1.83 | 46.479 | 8.325 | 12.664 | 3.358 | 16.021 | 12.85 | 12.472 | 2.023 |  |
|  |  |  |  |  |  |  |  |  |  |  |  |  |
|  | 72h postoperatively | group A (n=40) | 0.88 | 146.063 | 16.416 | 14.372 | 8.777 | 23.15 | 22.683 | 27.286 | 4.314 | 0.872 |
|  |  |  |  |  |  |  |  |  |  |  |  |  |
|  |  | group B (n=38) | 2.65 | 104.063 | 15.068 | 17.524 | 9.398 | 26.922 | 21.803 | 20.141 | 3.267 |  |
|  |  |  |  |  |  |  |  |  |  |  |  |  |
|  | 120h postoperatively | group A (n=40) | 0.48 | 116.096 | 6.881 | 8.161 | 3.447 | 11.608 | 10.388 | 18.43 | 2.914 | 0.660 |
|  |  |  |  |  |  |  |  |  |  |  |  |  |
|  |  | group B (n=38) | 1.46 | 26.947 | 7.367 | 5.427 | 5.652 | 11.08 | 9.006 | 5.815 | 0.943 |  |
|  |  |  |  |  |  |  |  |  |  |  |  |  |
|  |  |  |  |  |  |  |  |  |  |  |  |  |
| IL-6 | Preoperative | group A (n=40) | 1.5 | 22.86 | 4.52 | 3.575 | 2.555 | 6.13 | 5.307 | 4.144 | 0.655 | 0.987 |
|  |  |  |  |  |  |  |  |  |  |  |  |  |
|  |  | group B (n=38) | 1.5 | 22.76 | 4.155 | 3.963 | 2.15 | 6.113 | 5.323 | 4.741 | 0.769 |  |
|  |  |  |  |  |  |  |  |  |  |  |  |  |
|  | 24h postoperatively | group A (n=40) | 2.7 | 218.8 | 31.455 | 58.423 | 9.975 | 68.398 | 49.288 | 53.137 | 8.402 | 0.190 |
|  |  |  |  |  |  |  |  |  |  |  |  |  |
|  |  | group B (n=38) | 1.5 | 159.6 | 21.53 | 42.668 | 10.768 | 53.435 | 35.621 | 36.178 | 5.869 |  |
|  |  |  |  |  |  |  |  |  |  |  |  |  |
|  | 72h postoperatively | group A (n=40) | 1.5 | 151.7 | 7.81 | 8.213 | 5.07 | 13.283 | 13.537 | 23.666 | 3.742 | 0.893 |
|  |  |  |  |  |  |  |  |  |  |  |  |  |
|  |  | group B (n=38) | 1.5 | 48.16 | 11.53 | 10.998 | 6.57 | 17.568 | 14.105 | 10.81 | 1.754 |  |
|  |  |  |  |  |  |  |  |  |  |  |  |  |
|  | 120h postoperatively | group A (n=40) | 1.9 | 33.98 | 10.08 | 7.098 | 6.988 | 14.085 | 11.634 | 7.013 | 1.109 | 0.604 |
|  |  |  |  |  |  |  |  |  |  |  |  |  |
|  |  | group B (n=38) | 1.5 | 30.81 | 9.915 | 8.555 | 6.675 | 15.23 | 10.808 | 6.97 | 1.131 |  |
|  |  |  |  |  |  |  |  |  |  |  |  |  |

Notes: NE : Neutrophils; CRP: C reactive protein C; NLR: neutrophil-to-lymphocyte ratio; PLR: platelet-to lymphocyte ratio; MLR: monocyte to lymphocyte ratio; CLR: C-reactive protein to lymphocyte ratio; IL-6: Interleukin-6.
